# Infertility network and hub genes for nonobstructive azoospermia utilizing integrative analysis

**DOI:** 10.18632/aging.202559

**Published:** 2021-02-17

**Authors:** Baoquan Han, Zihui Yan, Shuai Yu, Wei Ge, Yaqi Li, Yan Wang, Bo Yang, Wei Shen, Hui Jiang, Zhongyi Sun

**Affiliations:** 1 Department of Urology, Peking University Shenzhen Hospital, Shenzhen Peking University, The Hong Kong University of Science and Technology Medical Center, Shenzhen 518036, China; 2 College of Life Sciences, Institute of Reproductive Sciences, Qingdao Agricultural University, Qingdao 266109, China; 3 Department of Urology, Zaozhuang Hospital of Zaozhuang Mining Group, Zaozhuang 277100, China; 4 Guangdong and Shenzhen Key Laboratory of Male Reproductive Medicine and Genetics, Institute of Urology, Peking University Shenzhen Hospital, Shenzhen PKU-HKUST Medical Center, Shenzhen 518036, China; 5 Department of Urology, Department of Andrology, Department of Human Sperm Bank, Peking University Third Hospital, Beijing 100191, China

**Keywords:** nonobstructive azoospermia, integrative analysis, scRNA-seq, male infertility, biomarkers

## Abstract

Non-obstructive azoospermia (NOA) is the most severe form of male infertility owing to the absence of sperm during ejaculation as a result of failed spermatogenesis. The molecular mechanisms of NOA have not been well studied. Here, we revealed the dysregulated differentially expressed genes in NOA and related signaling pathways or biological processes. Cluster features of biological processes include spermatogenesis, fertilization, cilium movement, penetration of zona pellucida, sperm chromatin condensation, and being significantly enriched metabolic pathways in proximal tubule bicarbonate reclamation, aldosterone synthesis and secretion, glycolysis and glycogenesis pathways in NOA using Gene Ontology analysis and pathway enrichment analysis. The NOA gene co-expression network was constructed by weighted gene co-expression network analysis to identify the hub genes (*CHD5* and *SPTBN2*). In addition, we used another Gene Expression Omnibus dataset (GSE45887) to validate these hub genes. Furthermore, we used the Seurat package to classify testicular tissue cells from NOA patients and to characterize the differential expression of hub genes in different cell types from different adult males based on the scRNA-seq dataset (GSE106487). These results provide new insights into the pathogenesis of NOA. Of particular note, *CHD5* and *SPTBN2* may be potential biomarkers for the diagnosis and treatment of NOA.

## INTRODUCTION

Infertility is a serious health problem that may be associated with aging [1]. It is one of the most commonly diagnosed conditions associated with either reproductive health or male-related problems; it is multifactorial and accounts for approximately half of all infertility cases [2]. However, the genetic basis of male infertility has not been extensively studied [3]. A significant portion of male infertility is associated with idiopathic azoospermia, usually manifested as non-obstructive azoospermia (NOA), which affects about 1% of all adult males [4]. Currently, NOA remains the most clinically severe form of male infertility due to the absence of sperm in the ejaculate as a result of failed spermatogenesis [5]. Several studies have reported the involvement of genetic factors in the occurrence of NOA, such as defective chromosome number, microdeletions of the Y-chromosome, and autosomal mutations or polymorphisms in multiple biological pathways [6, 7]. While there has been some progress regarding the etiology of the disease (involving either intrinsic testicular impairment or insufficient gonadotropin production), the molecular defects responsible for NOA that are associated with male sterility are largely unknown.

An adequate diagnosis of NOA is currently far from satisfactory, especially in terms of identifying molecular causes, which can be complex [8]. Therefore, the identification of potential biomarkers involved in NOA appears to be crucial. With the rise of high-throughput sequencing technology, several key NOA-related genes have been identified. In 2008, using genome-wide gene expression analysis, Hiroyuki et al. identified *ART3* as a susceptibility gene for NOA [9]. Subsequent study found that deletion or under-expression of the Y-chromosome genes *CDY2* and *HSFY* is associated with the blocking of sperm maturation in American men with NOA [10]. More recent studies have indicated that there are several potential NOA-related biomarkers (such as *IL1-RA, AKAP4, UBQLN3, CAPN11, GGN, SPACA4, SPATA3,* and *FAM71F1*) [8, 11]. Moreover, other researchers have identified *ENTPD6* and *STX2* as potential NOA pathogenic genes [12, 13].

Currently, except for traditional microarray analysis, several studies have used weighted gene co-expression network analysis (WGCNA) analysis [14], whole-exome sequencing [15], and single-cell transcriptome sequencing (scRNA-seq) [16, 17] to screen for novel infertility causative genes in NOA. The resulting data provide a basis for further studies on the pathogenesis of NOA, but these existing studies do not integrate multiple sequencing data, which makes the results obtained by a single analytical approach less convincing. Correlation networks are increasingly being used in bioinformatics applications, especially using WGCNA. This technique has been widely used to explore the large and complex relationships between microarray and RNA sequence data, which provides a convenient and effective solution for screening potential biomarkers for clinical prognosis and therapy [18, 19]. Single-cell transcriptome sequencing is an optimized second-generation sequencing technology that has been extensively developed and applied to biological and pathological research [20–22]. It can be used to study the functional status of individual cells, to infer and discover new cell types in an unbiased manner, and can also be used to construct differentiation trajectories for cell lineages, as well as molecular maps of cell developmental profiles. Since its development, single-cell transcriptome sequencing has been widely used in cancer and bio-developmental fields with excellent results. Although two studies performed single-cell transcriptome sequencing on testicular tissue from NOA patients [16, 17], no study has yet applied these open resources to an integrated analysis of NOA; this omission lays the foundation for our study.

In the current study, WGCNA and scRNA-seq were combined to analyze hub gene profiling of NOA samples extracted from the Sequence Read Archive (SRA) database, while two additional GEO databases were used to validate the results. After integrative analysis, two hub genes, *CHD5* and *SPTBN2*, were screened in the NOA group of patients. The results of this study will be informative for basic research on NOA and provide a theoretical basis for the clinical diagnosis and treatment of NOA.

## RESULTS

### Overview of the transcriptomes of NOA and GO enrichment analysis

In order to elucidate the molecular pathogenesis of NOA, we firstly screened the DEGs between normal testicular biopsies and NOA cases using the limma package and then built the volcano map and heat map using the ggplot2 and pheatmap packages. The heat map of all mRNAs is shown in Supplementary Figure 1A, where significant differences in gene expression between the two groups can be seen. Subsequently, the volcano map was constructed using differentially expressed mRNAs (|log2fold change|≥2, *p*-value < 0.05) as shown in Supplementary Figure 1B. In total, 214 mRNAs displayed the differential expressions in NOA, including two upregulated and 212 downregulated mRNAs (|log2fold change|≥2, *p*-value < 0.05) (Supplementary Table 1). In addition, from the heat map and the cluster dendrogram (Supplementary Figure 3A), it can be seen that the normal testicular biopsies and NOA cases were allocated into two distinct main clusters (control and NOA).

**Figure 1:**
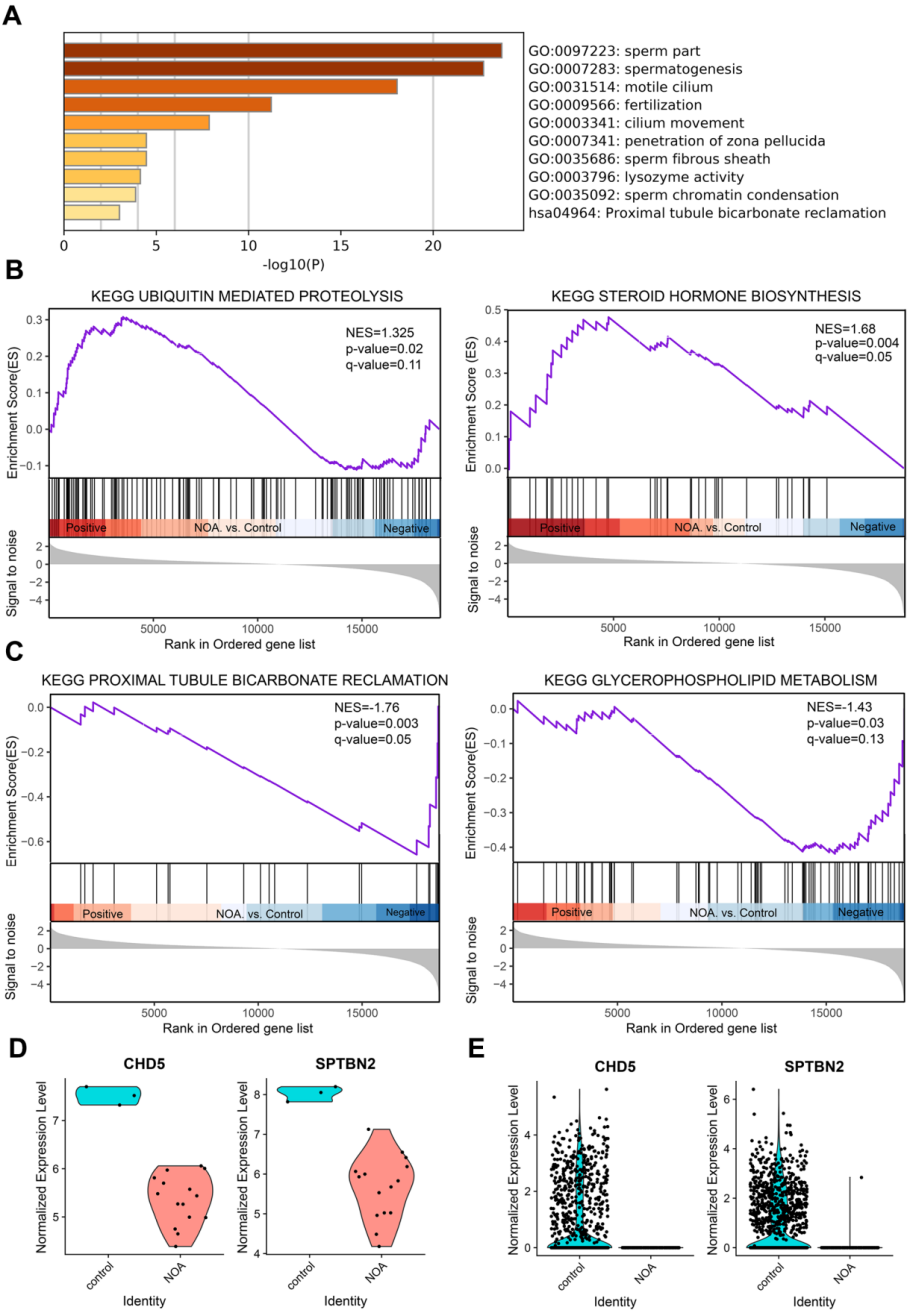
**GO enrichment and Gene Set Enrichment Analysis of NOA, and validation of hub genes.** (**A**) Top 10 clusters with their representative enriched term based on GO enrichment analysis of DEGs. (**B**) NOA samples were correlated positively with gene signatures related to Steroid Hormone Biosynthesis and Ubiquitin Mediated Proteolysis pathways. (**C**) NOA samples were correlated negatively with gene signatures related to Glycerophospholipid Metabolism and Proximal Tubule Bicarbonate Reclamation pathway. (**D**) The normalized expression validation of hub genes using the GSE45887 dataset. The Y-axis expressions were normalized by log_2_(TPM/10+1). (**E**) The normalized expression validation of hub genes using the GSE106487 dataset. The Y-axis expressions were normalized by log_2_(TPM/10+1).

After Metascape analysis, the Top 10 clusters with their representative enriched terms are shown in Figure 1A and Supplementary Table 2. GO analysis results were enriched in Biological Process, Cellular Components, and Molecular Functions. Biological Process clusters mainly include spermatogenesis, fertilization, cilium movement, penetration of zona pellucida, and sperm chromatin condensation. Cellular Components consist mainly of sperm part, motile cilium, and sperm fibrous sheath. For Molecular Functions, the main component is lysozyme activity. To further capture the relationships between the terms, a subset of enriched terms was selected and rendered as a network plot (Supplementary Figure 1C), where terms with a similarity > 0.3 were connected by edges. We selected the terms with the best *p*-values from each of the 20 clusters, with the constraint that there were no more than 15 terms per cluster and no more than 250 terms in total. From the network plot, the selected DEGs were closely related to spermatogenesis, fertilization, cilium movement, sperm part, motile cilium, and sperm fibrous sheath, further indicating the important role of DEGs in spermatogenesis-related bioprocesses. Additionally, KEGG analysis was conducted (Supplementary Table 3), and was mainly enriched in Proximal tubule bicarbonate reclamation, Aldosterone synthesis and secretion, Glycolysis/Gluconeogenesis, and Phosphonate and phosphinate metabolism. When combined with the above GO analysis, no pathways overlapped with the GO results.

### Gene set enrichment analysis of NOA

KEGG analysis based on the GSEA analysis method showed that the upregulated DEGs were significantly enriched in Steroid Hormone Biosynthesis and Ubiquitin Mediated Proteolysis pathways (Figure 1B), and the downregulated DEGs were enriched in Glycerophospholipid Metabolism and Proximal Tubule Bicarbonate Reclamation pathway (Figure 1C). When combined with previous data (Supplementary Table 3), the Proximal Tubule Bicarbonate Reclamation pathway may be of great importance in NOA pathogenesis.

### Protein-protein interaction of NOA

To further investigate all the DEGs (Supplementary Table 1) and the potential protein levels, the STRING database was applied to reveal the core PPI network. As shown in Supplementary Figure 2A, the core PPI network was constructed by molecular action (confidence threshold: 0.4), including 199 nodes and 297 edges. These selected DEGs were found to interact closely with biological processes or cellular components, including sexual reproduction (48 genes, nodes colored with green), spermatogenesis (38 genes, nodes colored with red), and sperm flagellum (14 genes, nodes colored with blue). Subsequently, we reconstructed the PPI networks of all DEGs by molecular action (confidence threshold: 0.7) for the above biological processes or cellular components (Supplementary Figure 2B) to compare the two methods. Based on a higher confidence threshold, there were only 37 edges, which indicated that an even higher confidence threshold could further narrow the positive target interaction. Of particular note, even using the higher confidence threshold, the *SPTBN2* gene still existed in PPI networks, which may suggest an underlying role for the *SPTBN2* gene in NOA pathogenesis.

### Weighted gene correlation network analysis of NOA

**Figure 2:**
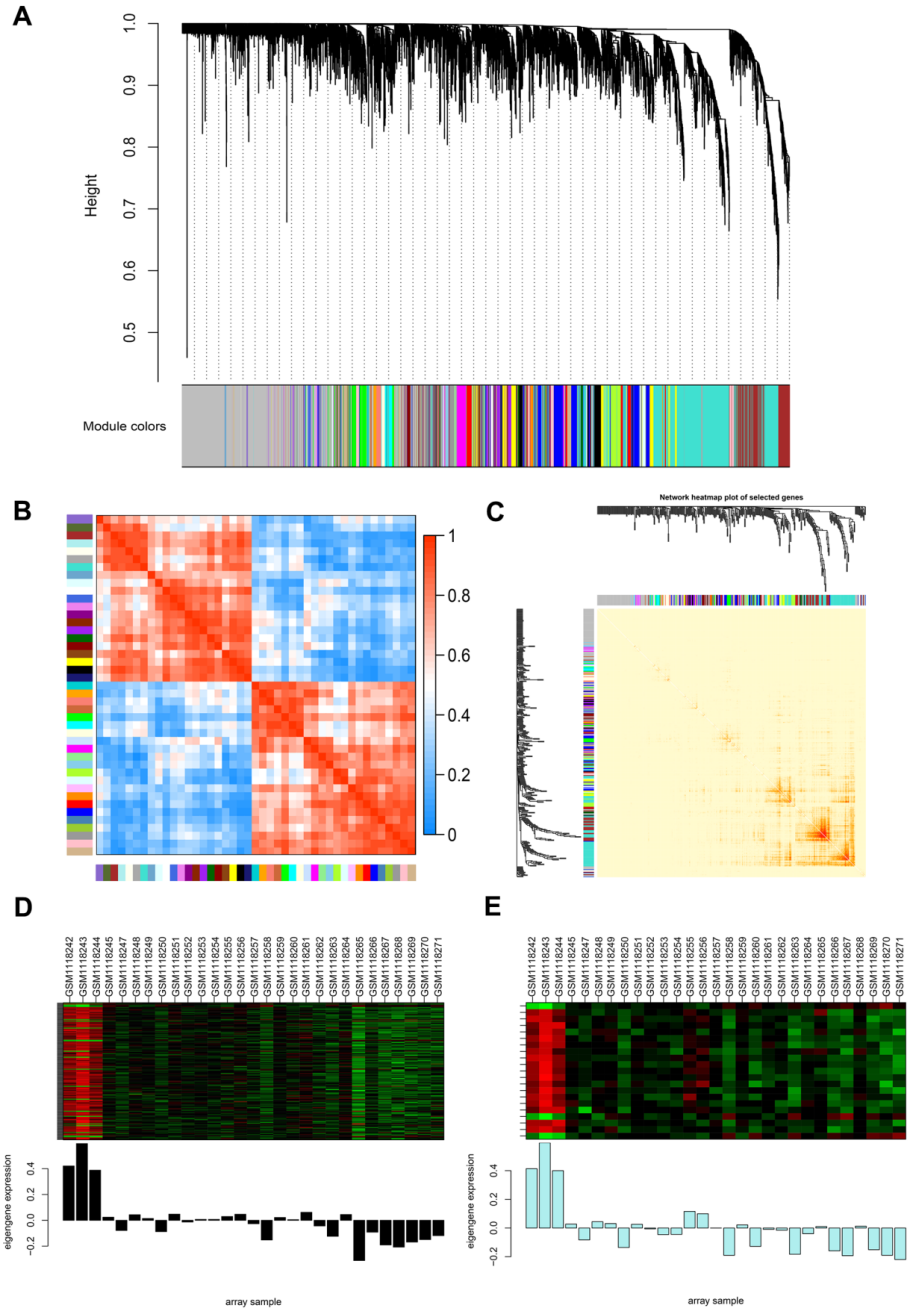
**Weighted gene co-expression network analysis (WGCNA) of genes between the control and NOA groups.** (**A**) Hierarchical cluster tree showing co-expression modules identified by WGCNA. The X-axis represents genes in different modules marked with different colors. (**B**) Heatmap plot of the adjacencies in the eigengene network. Each row and column in the heatmap correspond to one module eigengene (labeled by color). In the heatmap, the blue color represents low adjacency (negative correlation), while red represents high adjacency (positive correlation). Squares of red color along the diagonal are the meta-modules. (**C**) Heat map plot shows the topological overlap matrix (TOM) among 400 randomly selected genes. Light colors depict a small overlap, and the red color indicates a greater overlap. The left side and the top side show the gene dendrogram and module assignment. (**D**, **E**) Two modules with the highest relative rates were selected to localize the hub genes, they are separately colored with black (**D**) and pale turquoise (**E**). The Y-axis expressions were normalized by log_2_(TPM/10+1).

To precisely clarify the key modules and hub genes in NOA, WGCNA was used to identify clusters (modules) of highly correlated genes and correlation networks of NOA. GSM1118241 (control) and GSM1118246 (NOA) were excluded from the analysis after quality assessment (Supplementary Figure 3A). The power of β was set at 24 to ensure a scale-free network and the mean connectivity remained normal (Supplementary Figure 3B), which indicated a good scale-free topology index of our dataset analysis. Gene modules were calculated, and gray modules represent genes that cannot be clustered into any other modules (Figure 2A and Supplementary Figure 3C). Furthermore, 44 gene modules were identified by the hierarchical clustering dendrogram and Module eigengene adjacency heatmap (Figure 2A, 2B). The interactions between gene modules were then analyzed and a TOM plot of the gene network was generated based on the corresponding hierarchical clustering dendrogram and modules (Figure 2C).

### Identification and validation of the hub genes of NOA

As WGCNA generated a huge gene network, we narrowed two modules (Figure 2D, 2E) for the network construction to localize the hub genes using the limma package. As a result, 179 nodes and 3306 edges were screened and used for the PPI network construction by molecular action (confidence threshold: 0.9) for further analysis (Supplementary Figure 1E). The MCODE plugin of Cytoscape was used to screen three subclusters (Supplementary Figure 1E and Supplementary Table 4) and calculate K-core values of each subcluster. After that, we extracted subcluster 1 and used Cytoscape to construct the core network (Supplementary Figure 1D). From the above two networks (Supplementary Figure 1D, 1E), *CHD5* and *SPTBN2* were preliminarily screened as the hub genes in NOA patients. Subsequently, we cross-referenced the gene set of each subcluster set separately with the previous total DEGs set (|log2fold change|≥2, *p*-value < 0.05) to screen for its common component. Ultimately, we screened to obtain these two hub genes (*CHD5* and *SPTBN2*) with the highest differential expression in NOA patients.

In order to verify the accuracy of the differential expression of above two hub genes, we tested their expression using the other dataset; the results showed that the expression was significantly reduced in the GSE45887 dataset (Figure 1D). In addition, while the current study was being performed, Chen et al. used Whole-exome sequencing to compare the whole genomes of NOA patients with healthy people, and the results show that the *CHD5* gene is one of the infertility causative genes of NOA [15], which also demonstrates the reliability of the method used in this study.

### Integrative analysis of hub genes with single cell transcriptome

**Figure 3:**
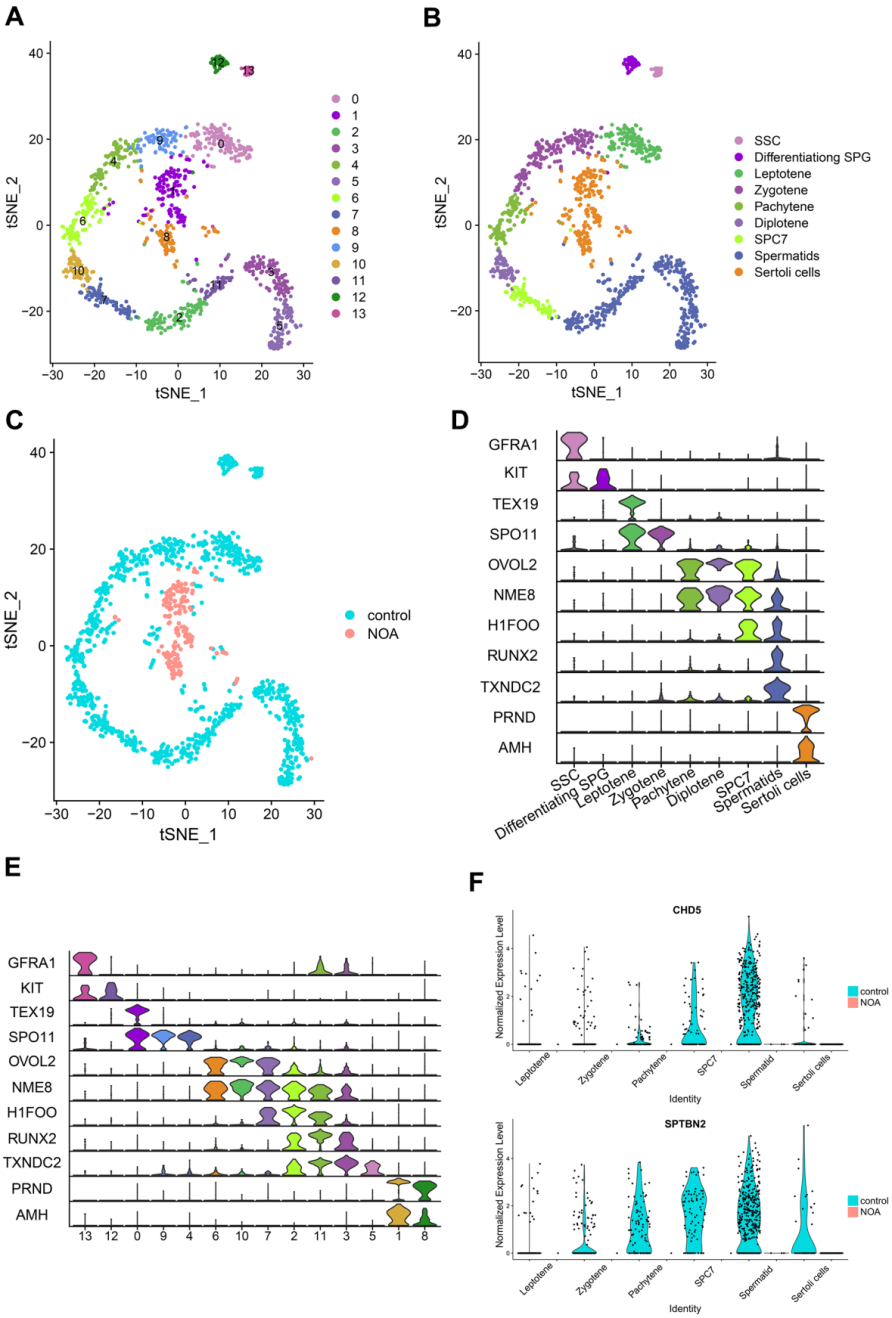
**Identification and comparison of different cell types in different samples based on the single-cell RNA-seq dataset.** (**A**) Cell cluster distribution in the TSNE plot. (**B**) Different types of cells and different samples identified in tSNE plots. SSC, spermatogonial stem cells; Differentiating SPG, differentiating spermatogonia; SPC7, cell mixtures including diakinesis, metaphase, anaphase, telophase, and secondary spermatocytes. (**C**) Comparison of different samples in tSNE plots. (**D**) Multiviolin plot of expressions of specific gene markers in identified cell types. X-axis represents diverse cell types. Y-axis represents the expression of specific gene markers. (**E**) Multiviolin plot of expressions of specific gene markers in different clusters. X-axis represents different clusters. Y-axis represents the expressions of specific gene markers. (**F**) The expression validation of hub genes from diverse cell types in the NOA group compared with the control group. The Y-axis expressions were normalized by log_2_(TPM/10+1).

In this section, we integrated the scRNA-seq dataset (GSE106487) with the hub genes obtained from a previous screen for integrated analysis to further clarify the differential expression of these genes in specific cell types. The scRNA-seq data were first clustered and identified into different cell types (Figure 3A–3C). During spermatogenesis, all cell types (Figure 3D) and clusters (Figure 3E) from sperm stem cells to spermatocytes were identified by specific genetic markers. From Figure 3B, 3C, more than 95% of the cells in the NOA group were identified as Sertoli cells. In addition, we compared gene expression in the control and NOA groups to further validate the findings of the microarray (Figure 1E). Furthermore, the expression of *CHD5* and *SPTBN2* was significantly down-regulated from different cell types in the NOA group compared to the control group (Figure 3F), which further indicated the potential role of these two hub genes in NOA pathogenesis.

## DISCUSSION

Unlike obstructive azoospermia (OA), the pathogenesis of NOA is more complex and difficult to understand [9]. To further investigate the molecular mechanism of the pathogenesis of NOA, we integrated three datasets from the SRA and GEO databases in this study and successfully identified two hub genes (*CHD5* and *SPTBN2*) that were significantly under-expressed in the testicular tissues of NOA patients. Furthermore, the differential expression of these two genes in different cell types of testicular tissues were elucidated in greater detail using single-cell transcriptome sequencing analysis. The above results confirmed that *CHD5* and *SPTBN2* are potential biomarkers for NOA diagnosis and treatment.

The previous paper roughly screened for differentially expressed genes that might be associated with NOA disease, but could not accurately qualify specific differentially expressed genes. As these results differed from many previous studies, the existing published data were integrated and analyzed in the current study. Firstly, the existing dataset (GSE45885) was screened for NOA core network and hub genes using WGCNA analysis; two hub genes, *CHD5* and *SPTBN2*, were successfully screened. Moreover, a recent study in 2020 [15] identified 20 new NOA candidate genes affecting 25 NOA patients by means of WES sequencing. Among these genes, *CHD5* was considered to be a strong candidate gene, which can also fully confirm the feasibility of the present method and the reliability of the results in this study. Subsequently, we integrated and analyzed the above two hub genes using single-cell transcriptome data. After completing the clustering of different types of cells in the testis, we accurately elaborated the differential expression of the above two hub genes in different types of cells, which provided important theoretical support for revealing the pathogenesis of NOA and further ensured the credibility of the results of this study.

The most common previously reported NOA pathogenic candidate genes include the following: ART3, CDY2, HSFY, IL1-RA, AKAP4, UBQLN3, CAPN11, GGN, SPACA4, SPATA3, FAM71F1, ENTPD6, STX2, TEX11, TEX12, TEX14, TEX15, CD133, CD24, GSG1, BRDT, CHD5, MCM9, MLH3, and ZFX [9–13, 15, 23–26], of which, Chromodomain helicase DNA-binding protein 5 (CHD5) has been identified as a master regulator of the histone-to-protamine chromatin remodeling process. CHD5 deficiency affects spermatogenesis, resulting in male infertility in mice with phenotypes ranging from sperm deficiency to reduced sperm count [27]. According to our current analysis (Supplementary Figure 2A), CHD5 was associated with the following biological processes: sexual reproduction and spermatogenesis. Furthermore, the expression of CHD5 was significantly down-regulated in the NOA group compared to the control group (Figure 1D, 1E). Taken together, these results suggest that CHD5 may have an important role in the pathogenesis of NOA. It is well known that premature testicular aging, which mainly results from spermatogenic dysfunction, is an important pathogenesis of infertility in elderly men [28]. While the function of CHD5 is related to spermatogenesis, the correlation between premature testicular aging and CHD5 still needs to be explored. This study further demonstrates the possible important role of CHD5 gene in the pathogenesis of NOA. With the exception of the CHD5 gene, our study also identified an additional hub gene, SPTBN2. SPTBN2 has been shown to regulate the glutamate signaling pathway by stabilizing the glutamate transporter protein EAAT4 on the surface of the plasma membrane. Mutations in this gene cause spinal cerebellar ataxia (SCA5), which is characterized by neurodegenerative changes, progressive motor incoordination, dysarthria, and oculomotor incoordination [29]. According to the PPI construction section of the current analysis, even using the higher confidence threshold, the SPTBN2 gene still existed in PPI networks, which may suggest an underlying role for SPTBN2 in NOA pathogenesis. Moreover, the expression of SPTBN2 was significantly down-regulated in the NOA group compared to the control group (Figure 1D, 1E), which implies a possible role for this gene in the pathogenesis of NOA. However, the SPTBN2 gene has not yet been reported as a candidate gene for NOA pathogenesis; therefore, this role needs further exploration. Our study improves and completes the panel of candidate genes for NOA pathogenesis and provides a theoretical basis for subsequent studies in this area. However, due to the lack of clinical samples, this study was not able to conduct a corresponding validation test.

In summary, the current study used integrative analysis to map the multi-gene composition of NOA’s sterility network and hub genes, and the results suggest that *CHD5* and *SPTBN2* can serve as potential biological targets for the clinical diagnosis and treatment of NOA.

## MATERIALS AND METHODS

As our study was based on a conjoint analysis of existing data and no additional patients were included, ethical approval was not required.

### Data collection and preprocessing

Four normal spermatogenesis control and 27 NOA case microarray expression matrix files were downloaded from the National Center for Biotechnology Information (NCBI) Sequence Read Archive with the ID GSE45885. In addition, two expression matrix files with the ID GSE45887 (including four control and 16 NOA cases) and GSE106487 (consisting of two adult normal males, seven OA males, and one NOA male patient) were also prepared for subsequent validation and integrative analysis. The clinical information of testicular samples in GSE45885, GSE45887, and GSE106487 was obtained from published literature [8, 11, 16].

### Differentially expressed gene screening

The limma package was used to determine differentially expressed genes (DEGs) between normal testicular biopsies and NOA cases under the threshold of *p*-value < 0.05: A linear model was simply fitted to the expression matrix and further analysis was then performed using empirical Bayes. Significant DEGs between different groups were identified using “|log2fold change|≥2” and “*p*-value < 0.05” as the judgment threshold. The ggplot2 package and pheatmap package in R were used to build the volcano map and heat map.

### GO enrichment analysis and pathway enrichment analysis

Metascape analysis (http://metascape.org) was performed to accomplish Gene Ontology (GO) analysis to depict the unique biological significance based on DEGs between different groups [30]. The Kyoto Encyclopedia of Genes and Genomes (KEGG) database was used to determine important pathways, and was performed in combination with two websites (https://david-d.ncifcrf.gov/home.jsp and http://kobas.cbi.pku.edu.cn/kobas3). The “*p*-value < 0.05” and the “|log2fold change|≥2” were used as the cutoff criteria for GO and KEGG enrichment analyses.

### Gene Set Enrichment Analysis

For traditional analysis using DNA microarrays, the common approach involves focusing on a handful of genes at the top and bottom of L (i.e., those showing the largest difference) to discern telltale biological clues, but this approach has a few major limitations. To overcome these analytical challenges, we used a method called Gene Set Enrichment Analysis (GSEA) that evaluates microarray data (GSE45885) at the level of gene sets. Following the standard procedure for GSEA analysis [31], we first converted the expression dataset from GSE45885 into the tab-delimited GCT format as follows: the first column displays the gene symbol, while the second column is labeled “NA.” We then populated the subsequent columns with expression values from each sample. Subsequent operations were carried out in full accordance with GSEA’s standard protocol (http://www.gsea-msigdb.org/gsea/).

### Protein-protein interaction network building

Differentially expressed mRNAs (|log2fold change|≥2, *p*-value < 0.05) were taken into the Search Tool for the Retrieval of Interacting Genes/Proteins (STRING). The confidence scores were set at 0.4, 0.7, and 0.9 in diverse analysis. Then the Gene network files were input into Cytoscape software and the Molecular Complex Detection (MCODE) plugin of Cytoscape was used to analyze the core modules of the protein-protein interaction (PPI) network.

### Co-expression network construction

Correlation networks are increasingly being used in bioinformatics applications. In this part, WGCNA was conducted to uncover the correlation among genes using the WGCNA package. Firstly, expression data of DEGs (*p*-value < 0.05) was input into R software to inspect good genes and samples; GSM1118241 (control) and GSM1118246 (NOA) were excluded from the analysis after quality assessment. To ensure that the network was scale-free, the power of the β was set to 24. The minimum number of modules was 10. Hierarchical clustering dendrogram summarized the gene modules of different colors. Heat map and topological overlap matrix (TOM) plots were used to visualize the module structure. The threshold for output to Cytoscape was set to 0.02.

### Hub gene selection and validation

First, the gene network files exported from the WGCNA analysis were input into the Cytoscape software. The K-core values for each subcluster were then calculated using the MCODE plugin of Cytoscape. Referring to the detailed information of the GSE45887 dataset and previous studies [8, 11, 14], we confirmed that the sample of the GSE45887 dataset was different from that in the GSE45885 dataset, so the expression of hub genes was then validated using the GSE45887 dataset.

### Integrative analysis of hub gene with single-cell transcriptome

After the above validation, we conducted an integrative analysis using the single-cell RNA-seq dataset (GSE106487). For this dataset, we used the Seurat package to analyze cell transcriptome data including two control samples and one NOA sample. First, cells with less than 2000 genes and less than 10,000 transcripts were filtered and the expression of other cells was normalized using log_2_ (TPM/10+1). The data were then integrated, scaled, and centered using the ScaleData function. After PCA analysis, cells were clustered using the tSNE method with the resolution set to 1. Then, all clusters were identified as different cell types by specific genetic markers, respectively. After cell type identification, we chose the same cell type from testicular tissues of healthy and NOA adult males to further characterized the differential expression of the hub genes screened in the previous step using the violin plot.

### Data availability statement

The authors confirm that the data supporting the findings of this study are available within the article and its supplementary materials.

## Supplementary Material

Supplementary Figures

Supplementary Table 1

Supplementary Table 2

Supplementary Table 3

Supplementary Table 4
