# Supplementary material for: Infertility network and hub genes for nonobstructive azoospermia utilizing integrative analysis
Source: Aging (Albany NY). 2021 Feb 17;13(5):7052–66. doi: 10.18632/aging.202559 (PMC7993690; doi:10.18632/aging.202559)
Supplement: Supplementary Figures [file aging-13-202559-s001.pdf]

SUPPLEMENTARY FIGURES

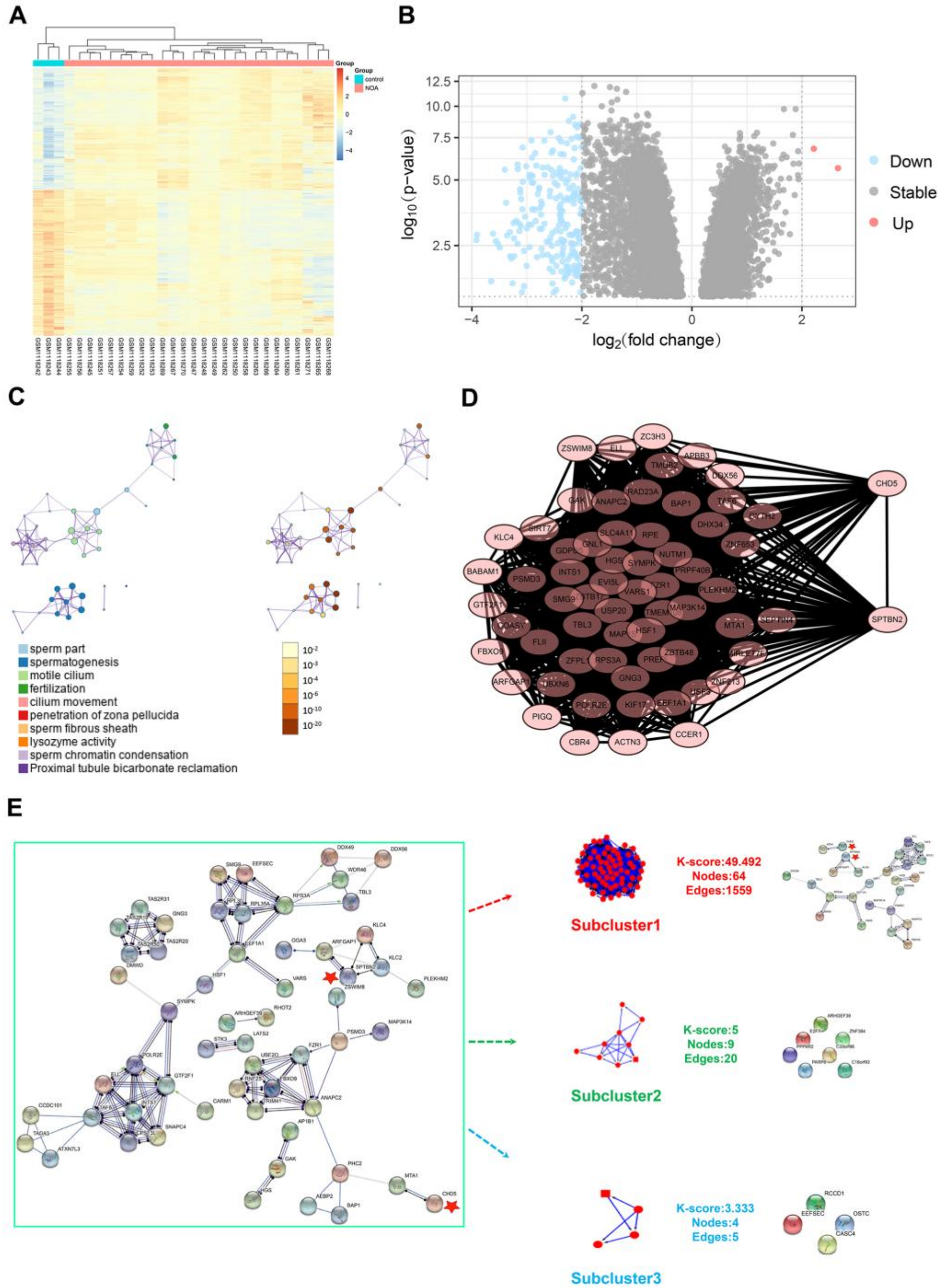

Supplementary Figure 1. The summary of DEGs, the PPI network of 181 selected DEGs, GO enrichment analysis, and the subcluster screen using the MCODE plugin of Cytoscape. (**A**) Heat map of all mRNAs detected by microarray. (**B**) The volcano plot of DEGs: 214 DEGs (|log2fold change|≥2, *p*-value < 0.05) were plotted in volcano plots with different colors, including two upregulated (red) and 212 downregulated mRNAs (blue). (**C**) Enriched Ontology Clusters whose nodes are colored by Cluster ID (left panel) and *p*-value (right panel). The terms were selected with the best *p*-values from each of the 20 clusters, with the constraint that there were no more than 15 terms per cluster and no more than 250 terms in total. (**D**) The core network of subcluster 1 was constructed using Cytoscape. (**E**) Three subclusters were screened based on the gene network files exported from the WGCNA analysis using the MCODE plugin of Cytoscape. The K-core values for each subcluster are displayed and the *CHD5* and *SPTBN2* genes are marked with red stars.

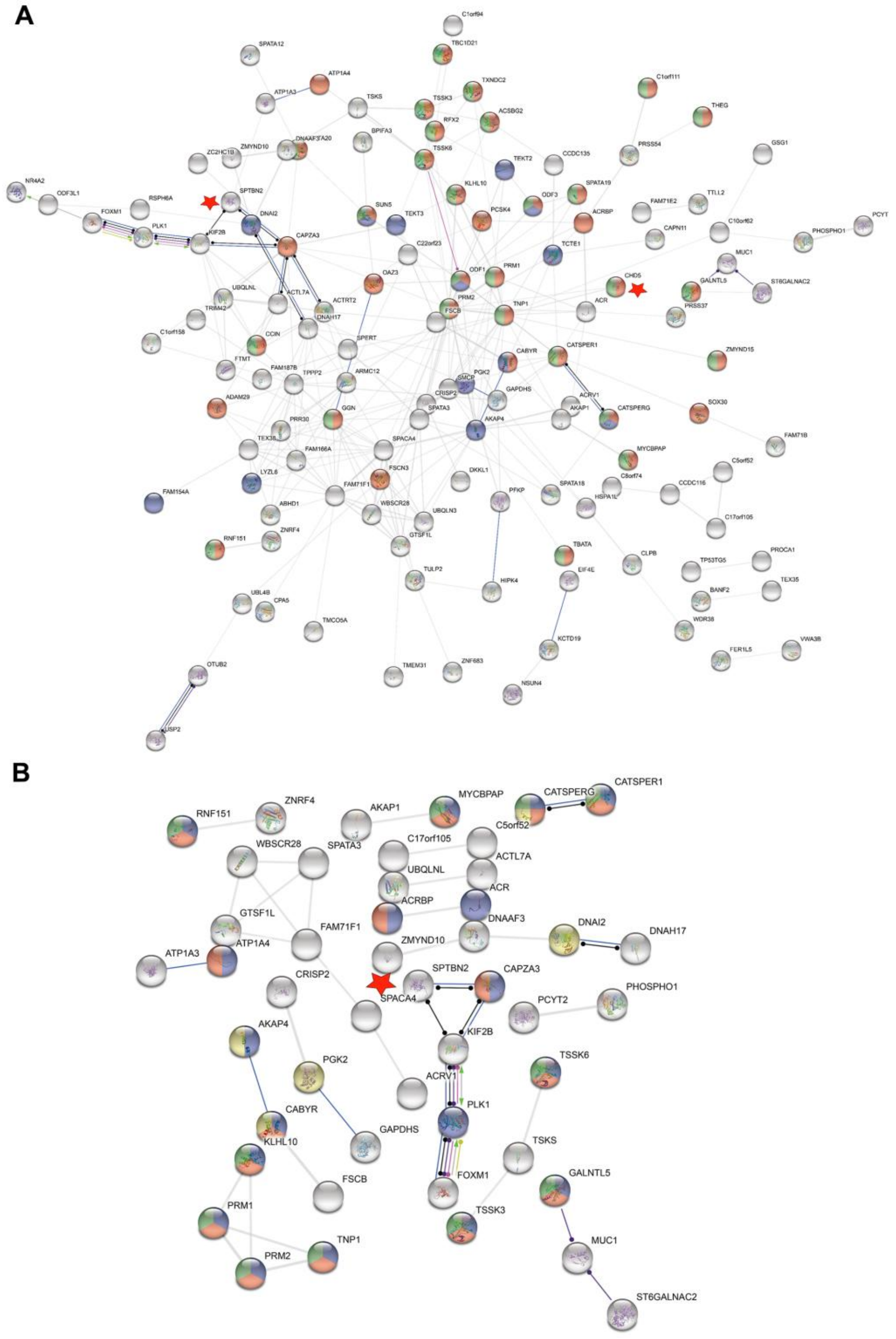

Supplementary Figure 2. The PPI networks based on all DEGs between control and NOA groups using different parameter settings. (**A**) The PPI network was constructed by STRING based on DEGs (|log2fold change|≥2, *p*-value< 0.05). Therein, the core PPI network was constructed by molecular action (confidence threshold: 0.4), including 199 nodes and 297 edges. These selected DEGs were found to interact closely with biological processes or cellular components, including sexual reproduction (48 genes, nodes are colored green), spermatogenesis (38 genes, nodes are colored red) and sperm flagellum (14 genes, nodes are colored blue). The *CHD5* and *SPTBN2* genes are marked with red stars. (**B**) The PPI network was constructed using STRING based on DEGs (|log2fold change|≥2, *p*-value< 0.05). PPI networks of all DEGs were reconstructed by molecular action (confidence threshold: 0.7) for the above-mentioned biological processes or cellular components to compare the difference between the two methods. Based on a higher confidence threshold, there were only 37 edges. The *SPTBN2* gene is marked with a red star.

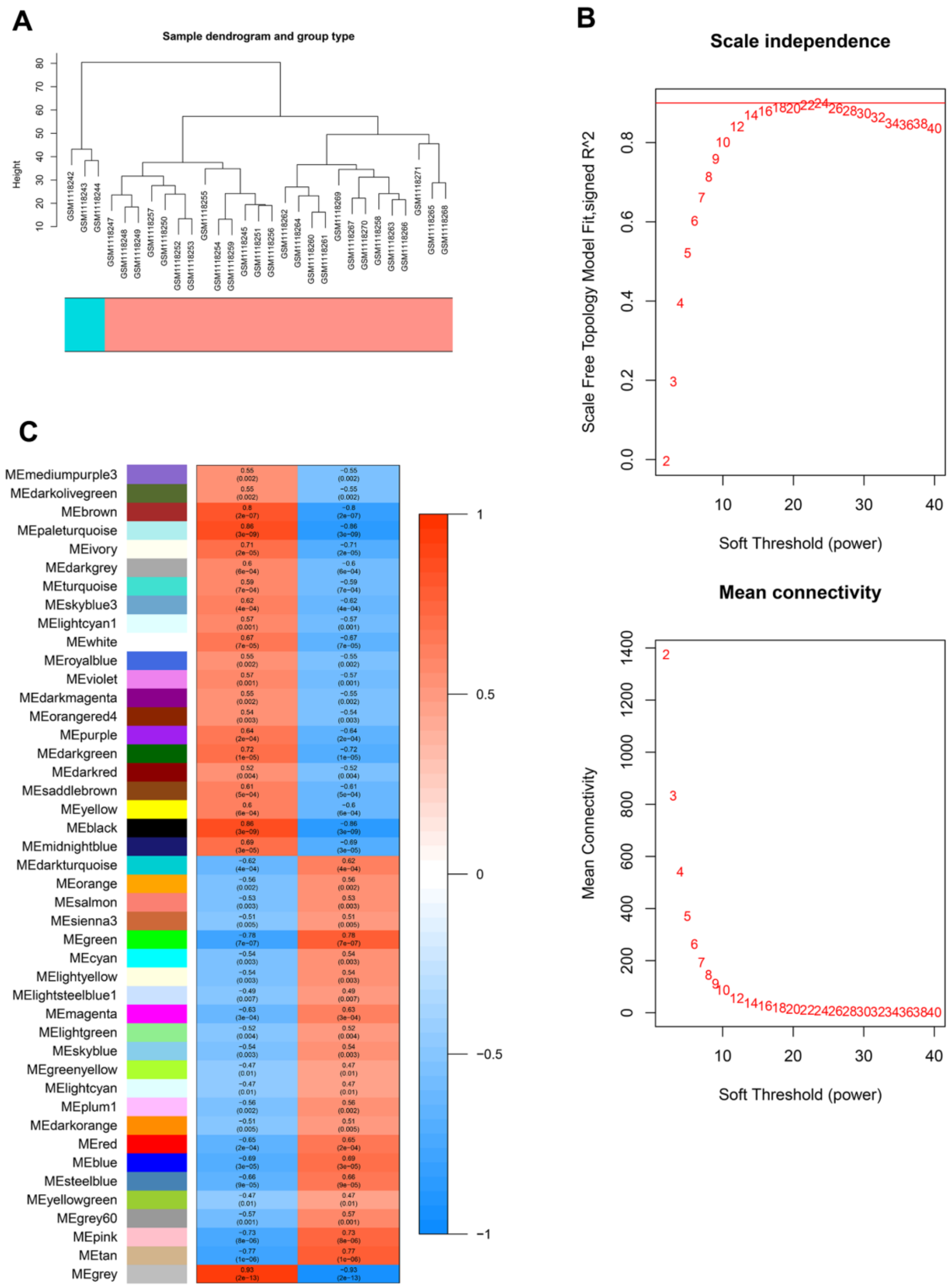

Supplementary Figure 3. The dendrogram, evaluation and the module trait relationships based on WGCNA analysis of genes between the control and NOA groups. (**A**) Cluster dendrogram displays the relationship between different samples. The different groups are marked with different colors presented at the bottom. (**B**) The evaluation of WGCNA of genes between the control and NOA groups. Top panel, analysis of the scale-free fit index for various soft thresholding powers (β). The red line parallel to the x-axis represents a scale-free fit index of 0.9; bottom panel, analysis of the mean connectivity for various soft-thresholding powers. X-axis represents soft-thresholding power. Y-axis represents mean connectivity. (**C**) The module trait relationships based on WGCNA analysis of genes between the control and NOA groups. Gene modules were calculated, and the gray module represents genes that cannot be clustered into any other modules. The different modules are marked with different colors.
